# Critical Reflections on Gentamicin Saline Irrigation for Appendectomy Wound Care

**DOI:** 10.1002/hsr2.71765

**Published:** 2026-01-16

**Authors:** Raza ur Rehman Rana, Rida Fatima, Shanza Arif

**Affiliations:** ^1^ Shaikh Khalifa Bin Zayed Al‐Nahyan Medical College, Shaikh Zayed Postgraduate Medical Institute (SZPGMI) Lahore Pakistan

I thoroughly read the paper by Bibek Shrestha et al. titled “Comparison of Gentamicin Saline Solution and Normal Saline in Reducing Surgical Site Infections in Open Appendectomy: A Randomized Controlled Trial” with keen interest. This research solves a clinically significant problem in surgical procedures, especially in resource‐constrained situations where open appendectomy is a frequent occurrence and affordable measures hold significance. Through a stringent comparison of the efficacy of gentamicin irrigation and normal saline, the paper adds meaningful evidence not only to surgical infection control practices but also emphasizes the need to focus on modifiable patient risk factors, like smoking and BMI. This paper is notable for providing a focus on pragmatic infection prevention in situations where every measure has to be a balance of efficacy, affordability, and safety. Although this paper presents strong evidence supporting a prospective role of gentamicin irrigation, design problems are noteworthy to give a fuller picture. Concerns include sample size assumptions, baseline imbalance, limited blinding, restriction to uncomplicated cases, and a narrow definition of surgical site infections, all of which limit robustness and generalizability.

Firstly, the sample size calculation for this study assumed gentamicin irrigation would reduce SSIs from 18% to 5%, representing about a 72% reduction. This expectation was overly ambitious and not backed by prior evidence. The actual reduction observed was much smaller (17.9% vs. 12.6%), leaving the study statistically underpowered to detect a realistic effect size. An underpowered study raises the risk of a Type II error, where a clinically meaningful benefit may go unnoticed. This shortcoming weakens the reliability of the conclusion that gentamicin irrigation offers no advantage. It is well established that unrealistic assumptions in sample size calculations often result in underpowered trials, increasing the likelihood of missing real treatment effects [1]. Conventional methods of estimating sample sizes also tend to overlook clinically realistic effect sizes, which can lead to misleading negative conclusions when differences are not detected [2]. Although the study was randomized, the baseline characteristics showed a statistically significant difference in Alvarado scores between the two groups (*p* = 0.045). Since the Alvarado score reflects disease severity, higher scores may predispose patients to a greater risk of infection. This imbalance suggests that randomization did not fully succeed in creating comparable groups, introducing potential confounding. According to CONSORT reporting standards, baseline comparability is crucial, as significant imbalances can bias outcomes even in randomized trials [3]. Furthermore, randomized studies that do not address stratification or block design imbalances risk producing misleading results due to hidden confounders [4].

Thirdly, the trial was single‐blinded, with patients unaware of their group allocation, but surgeons knew whether gentamicin or saline was being used. This awareness could have influenced intraoperative practices, such as wound handling, irrigation volume, or postoperative care, introducing performance bias. While blinding is often difficult to achieve in surgical trials, the lack of observer blinding in assessing infection outcomes raises additional concerns about detection bias. Inadequate blinding is a well‐recognized source of bias, as it may affect clinical decisions and undermine trial validity [5]. Nonpharmacologic trials, especially surgical ones, are particularly prone to such bias when both providers and outcome assessors are unblinded [6]. Fourthly, the study did not include patients with complicated appendicitis (e.g., perforated appendix, abscess, or generalized peritonitis), who face a higher risk of surgical site infections. While this ensured a more uniform study population, it greatly limits how broadly the findings can be applied. The question of whether antibiotic irrigation reduces infection risk is most relevant in complicated cases, where prevention matters most. Current surgical guidelines note that complicated appendicitis carries the highest infection risk, making it the key group for SSI prevention strategies [7]. Additionally, recent reviews show that uncomplicated cases usually recover well, whereas complicated cases account for most of the morbidity from SSI [8]. By excluding these patients, the study may underestimate the actual benefit or potential harm of gentamicin irrigation in real‐world surgical practice. Finally, the study identified only surface‐level surgical site infections, with no cases of deep or organ‐space infections. This is unusual in an appendectomy population, where deeper infections and intra‐abdominal abscesses are relatively common. The absence of such findings may reflect under‐detection due to short follow‐up or limited diagnostic rigor. According to CDC definitions, distinguishing between superficial, deep, and organ‐space infections is essential, and failing to capture all categories risks underestimating the true infection rate [9]. A comprehensive evaluation of surgical complications requires systematic detection of both superficial and deeper infections to reflect the overall burden accurately [10]. By focusing only on superficial SSIs, the study may have underestimated postoperative morbidity and reduced the accuracy of outcome measurement.

In conclusion, although this study adds worthwhile evidence about gentamicin irrigation during appendectomy, its methodological shortcomings such as underpowering, baseline imbalance, incomplete blinding, restriction to uncomplicated cases, and superficial‐only outcome measurement reduce the strength of its conclusions. Future trials should be larger, multicenter, and adequately powered, with inclusion of complicated appendicitis, extended follow‐up, and thorough evaluation of all categories of surgical site infection. Incorporating blinded outcome assessment and analyzing cost‐effectiveness alongside antimicrobial resistance patterns will provide stronger and more clinically relevant evidence to guide infection prevention in surgical practice.

## Author Contributions

**Raza ur Rehman Rana:** methodology, visualization, writing – review and editing. **Rida Fatima:** conceptualization, methodology, writing – original draft. **Shanza Arif:** writing – review and editing.

## Funding

The authors received no specific funding for this work.

## Ethics Statement

This is a Comment article providing a critique of a previously published study and did not involve human participants or new data collection.

## Consent

The authors have nothing to report.

## Conflicts of Interest

The authors declare no conflicts of interest.

## Declaration of Generative AI and AI‐Assisted Technologies in the Writing Process

After the preparation of this Letter, the author used OpenAI's ChatGPT in order to enhance the readability and language of the manuscript. After using this tool, the author reviewed and edited the content as needed and takes full responsibility for the content of the publication.

## Data Availability

The authors have nothing to report.
